# Recent Advances in Shockwave Therapy for Musculoskeletal and Soft-Tissue Disorders

**DOI:** 10.3390/life15121912

**Published:** 2025-12-13

**Authors:** Larisa Ryskalin, Federica Fulceri, Maria Cristina D’Agostino, Mario Vetrano, Maria Chiara Vulpiani, Marco Gesi

**Affiliations:** 1 Department of Translational Research and New Technologies Medicine and Surgery, University of Pisa, Via Roma 55, 56126 Pisa, Italy; larisa.ryskalin@unipi.it (L.R.); federica.fulceri@unipi.it (F.F.); 2 Shock Waves Center, Ortho-Rehabilitation Department IRCCS Humanitas Research Hospital, 20048 Rozzano (MI), Italy; cristina.dagostino@humanitas.it; 3 Physical Medicine and Rehabilitation Unit, Sant’Andrea Hospital, Sapienza University of Rome, 00189 Rome, Italy; mario.vetrano@uniroma1.it (M.V.); mariachiara.vulpiani@uniroma1.it (M.C.V.)

Extracorporeal shockwave therapy (ESWT) represents a form of “mechanotherapy” that operates by delivering acoustic waves to biological tissues. From a biophysical standpoint, shockwaves exert mechanical forces that stimulate a cascade of biological signaling events, ultimately promoting tissue regeneration and healing through “mechano-transduction” [1,2,3,4]. Originally employed in the 1980s for its disintegrative effects on urinary stones (lithotripsy), shockwave technology has since undergone remarkable development, with clinical applications across a wide spectrum of medical specialties, particularly in the management of musculoskeletal and soft-tissue disorders. Pioneering studies in the early 1990s demonstrated the successful use of shockwave therapy for nonunion long-bone fractures, plantar fasciitis, lateral epicondylitis (tennis elbow), and calcific rotator cuff tendinitis. Since then, ESWT has garnered increasing attention as a growing body of research has revealed its potential to yield a variety of beneficial therapeutic effects on the locomotor system, extending way beyond purely mechanical, disintegrative impacts. Nowadays, ESWT is considered a non-invasive, well-tolerated, and safe therapeutic option in orthopedics, offering promising outcomes in pain relief, tissue healing, and functional recovery [5,6,7,8,9,10,11,12]. Current evidence supports a complex interplay of ESWT-induced mechanisms, including enhanced tissue perfusion and neoangiogenesis, immunomodulatory effects, fibroblast stimulation, stem cell activity regulation, and analgesia [13,14,15,16,17,18,19,20]. At the same time, emerging findings show that ESWT can induce the release of extracellular vesicles (EVs), especially exosomes, representing a major breakthrough in EV-based regenerative medicine [21,22,23,24]. This mechanism holds significant promise for the treatment of various musculoskeletal injuries and may ultimately position ESWT as a cutting-edge, non-invasive treatment modality for modulating cell-to-cell communication and enhancing tissue regeneration.

While significant advances have been made in the clinical application of ESWT, the precise cellular and molecular mechanisms underlying the therapeutic effects of shockwaves remain subjects of intense ongoing research. At the same time, the development of different types of shockwave generators further complicates this issue, as each type of shockwave device (i.e., electrohydraulic, piezoelectric, and ballistic) features a different energy signature and bioeffects, potentially leading to variations in treatment outcomes. Furthermore, methodological inconsistencies in treatment protocols across studies (e.g., shockwave device, number of sessions, day intervals between sessions, number of impulses, and energy levels) add another layer of complexity, making it difficult to establish a universally accepted “gold standard” for treatments. This, in turn, represents a compelling opportunity for future research, as identifying optimal treatment parameters could significantly enhance patient outcomes and further advance the clinical effectiveness of ESWT.

This Special Issue aimed to collect experimental and clinical evidence highlighting the latest advances in the application of ESWT to treat musculoskeletal and soft-tissue disorders, combining original research with review articles addressing the key aspects of this emerging field.

Yoon et al. [25] conducted an open-label, randomized, controlled trial to evaluate whether focused ESWT is a safe adjunctive therapy to promote healing in elderly patients with iatrogenic abductor muscle injury following intramedullary nailing for hip fractures. While the functional improvements were not statistically significant, early postoperative ESWT was shown to accelerate pain reduction, which may accelerate mobilization and rehabilitation. From a clinical standpoint, this study suggests that ESWT could serve as a non-pharmacological, safe pain relief modality that enhances recovery after hip fracture in geriatric patients. This, in turn, aligns with modern perioperative care to minimize opioid use and facilitate early mobilization.

In a prospective randomized clinical trial, Covelli et al. [26] evaluated the clinical and functional outcomes of focused ESWT compared to therapeutic exercise over 6 months in 72 patients with rhizarthrosis (i.e., osteoarthritis of the trapeziometacarpal joint of the hand). While both approaches were effective in managing these patients, ESWT provided more sustained clinical benefits, with more persistent improvements at the 6-month follow-up. These findings represent a significant advancement in the management of rhizarthrosis, as to date, only one previous clinical study has explored the use of ESWT for this condition [27]. In particular, this study demonstrated that ESWT has superior effects over hyaluronic acid infiltration in terms of pain reduction, which is consistent with the analgesic and anti-inflammatory effects of ESWT.

Santilli et al. [28] developed an artificial neural network (ANN) model to identify predictive factors influencing the clinical success of focused ESWT for chronic non-calcific supraspinatus tendinopathy. In particular, the study analyzed demographic and clinical variables, including age, gender, VAS, and Constant–Murley, Roles, and Maudsley scores, to predict minimal clinically successful therapy (MCST), defined as a ≥40% reduction in VAS, six months after ESWT. The ANN model demonstrated good predictive ability and discrimination, with a sensitivity of 80.7% and an area under the ROC curve of 0.701. Using the ANN, the authors demonstrated a more effective response to ESWT when the main predictive factors, expressed through clinical and functional scales, were better and the patients were male or younger. Furthermore, age before ESWT emerged as the most influential factor for achieving clinical success, which is in line with the existing literature showing greater ESWT responsiveness in younger individuals [29,30]. Conversely, patients who were older, female, or presented with poor initial clinical scores were less likely to achieve MCST, suggesting that the use of combined therapies would be more appropriate. Therefore, the study emphasized that patients’ characteristics and clinical scales should not only be considered as evaluation tools to verify improvements but also as prognostic factors that could be integrated in an ANN-based predictive model to facilitate decision-making for ESWT application.

Müller-Ehrenberg et al. [31] reviewed the mechanisms, applications, and effectiveness of ESWT in the management of myofascial pain syndrome (MPS). While the review highlights the moderate to good clinical efficacy of ESWT, it also reveals substantial methodological inconsistencies across the existing literature that limit definitive conclusions. Key shortcomings include inconsistent diagnostic criteria for MPS, wide variability in shockwave application parameters, markedly short and heterogeneous follow-up periods, and frequent nonadherence to the International Society for Medical Shockwave Treatment (ISMST) guidelines. Furthermore, another critical concern that emerged from the study is the frequent conflation of radial pressure wave (RPW) therapy with focused extracorporeal shockwave therapy (fESWT), despite their fundamental differences in generation devices, physical characteristics, and mechanisms of action. In light of these considerations, the authors call for future higher-quality, rigorously designed studies that employ standardized protocols to generate robust scientific evidence and advance ESWT as a reliable non-invasive treatment modality for MPS.

Lastly, this Special Issue also features an author response written by Covelli et al. [32], which further clarifies methodological concerns raised in a previous publication, thus contributing to the evolving dialog surrounding ESWT research.

In conclusion, this Special Issue perfectly reflects the dynamic and evolving landscape of ESWT while emphasizing the need for continued investigation to address the remaining questions surrounding its mechanisms of action. At the same time, it also highlights the need for further research to expand the clinical evidence, which will undoubtedly help refine treatment protocols and uncover new medical fields for shockwave application to treat both common and complex conditions.
